# Improvement in "Jumping Stump" Syndrome Following Diagnostic Sciatic Nerve Block and Home Exercise Program in a Transtibial Amputee

**DOI:** 10.7759/cureus.42278

**Published:** 2023-07-21

**Authors:** David Weinberg, Bradley Tucker

**Affiliations:** 1 Physical Medicine and Rehabilitation, Hospital of the University of Pennsylvania, Philadelphia, USA

**Keywords:** below-knee-amputation, sciatic nerve block, myoclonus in amputee, jumping stump syndrome, transtibial amputation

## Abstract

"Jumping Stump" syndrome is a rare postoperative complication seen in the residual limb of amputees, with only a few cases documented in the literature. It has been defined as a peripherally induced movement disorder leading to either dystonia, myoclonus, tremors, or choreiform movements in the amputated residual limb. It is often associated with significant discomfort and an inability to ambulate with a prosthetic limb. Treatment options remain inconclusive at this time.

We present a case of "Jumping Stump" syndrome in a young female transtibial amputee following revision transtibial amputation (TTA) with myodesis and targeted muscle reinnervation. About six weeks after revision surgery, the patient started experiencing significant myoclonus of the right residual limb when extending the knee. She was trialed on various oral pharmacologic agents over six months and had multiple prosthetic adjustments without any symptomatic relief. Moreover, the patient was also prescribed a daily knee range of motion (ROM) and stretching program. Six months after symptom onset, she underwent a diagnostic right sciatic nerve block and right biceps femoris point block with immediate and significant improvement in symptoms. She had a greater ROM in the affected limb without myoclonus and was able to ambulate once again with her prosthetic limb. Our patient’s response to a diagnostic nerve and motor point block, as well as her marked improvement of symptoms with a consistent home exercise (stretching) program, suggests that desensitization of a muscle-tendon stretch response likely accounted for the improvement of symptoms. It is hypothesized that chemodenervation via botulinum toxin, in addition to the consistent home stretching program, would have accelerated the improvement of symptoms and should be further explored as a potential treatment modality for "Jumping Stump" syndrome.

## Introduction

"Jumping Stump” syndrome is a rare complication seen postoperatively in amputees [1]. It has been defined as a peripherally induced movement disorder leading to either dystonia, myoclonus, tremors, or choreiform movements in the amputated residual limb [1,2]. Although its pathophysiology is unclear, it has been suggested that postoperative nerve damage can alter the afferent sensory neuron input to the central nervous system (CNS), leading to an abnormal efferent motor response [2]. Neuromas have also been implicated as a potential ectopic trigger zone in causing "Jumping Stump" syndrome, with case reports documenting symptomatic relief following neuroma excision [3-5].

One of the major functional complications of "Jumping Stump" syndrome is significant discomfort with prosthetic limb wearing. The resulting dystonia, tremors, or myoclonus in the residual limb can necessitate frequent prosthetic limb readjustments and difficulty with ambulation, causing significant psychosocial distress and repeated visits to physician offices. Ultimately, the symptoms can result in prosthetic abandonment, severely impacting the individual's mobility and ability to perform activities of daily living (ADLs).

In the literature, the onset of "Jumping Stump" syndrome varies significantly -- from a few weeks postoperatively to up to three years following amputation [2,3,5]. To our knowledge, no case has been documented following revision surgery with myodesis and targeted muscle reinnervation (TMR). Prior case reports, which have described various treatment modalities, have generally shown minimal or no symptomatic relief for patients. The treatments, which are commonly used for the management of neuropathic pain and spasticity, include therapy with baclofen, diazepam, pregabalin/gabapentin, and pramipexole [1,2,6]. One case report in 2016 [6] demonstrated a successful, but temporary, reduction in symptoms with botulinum neurotoxin type A injections, while another from 2011 [7] revealed partial improvement with pramipexole.

## Case presentation

The patient was a 27-year-old female with a prior right foot Lisfranc dislocation complicated by compartment syndrome that had required multiple fasciotomies. The patient experienced significant discomfort following surgery, resulting in decreased ankle mobility, persistent pain, and contracture development. One year after the initial Lisfranc dislocation, the patient underwent a transtibial amputation (TTA) with TMR of the tibial and deep peroneal nerves, regenerative peripheral nerve interface (RPNI) for the superficial peroneal nerve, and tenodesis of the soleus to the tibia. The immediate postoperative course was uncomplicated; however, three months following the initial TTA, the patient presented with persistent residual stump pain and palpable point tenderness. A neuroma resection of the right deep peroneal nerve was performed, which provided only mild symptom relief. Following the neuroma resection, the patient continued to exhibit moderate stump pain, which was exacerbated when ambulating in a prosthetic limb using a walking test socket. The patient subsequently underwent revision surgery approximately one year after the initial amputation, which included fibular shortening, beveling of the tibia, and revision myodesis along with revised TMR for the common peroneal nerve. Both revision surgeries were performed under general anesthesia without epidural or spinal injections.

About six weeks following revision surgery, the patient started to experience involuntary movements of her right residual limb when extending her knee, causing significant discomfort. Upon evaluation by a physiatrist, a physical exam was notable for the myoclonus of her hamstrings, provoked by knee extension. Myoclonus initially started at approximately 40 degrees of passive knee flexion and became forceful after achieving 30 degrees of knee flexion, severely limiting the patient's range of motion (ROM). At the same time, the patient would also experience concomitant myoclonus of her quadriceps--both of which were very discomforting to the patient. Passive flexion of the knee beyond 50 degrees would cease the myoclonus. The passive ROM of the knee was eventually limited to about 10 degrees of flexion due to significant discomfort. Active knee extension ROM was limited to 30 degrees of flexion given the onset of myoclonus and discomfort. On physical exam, it appeared that the hamstring muscles were the initiator of the myoclonus. Of note, the patient had normal patella reflexes bilaterally.

Over the subsequent months, the patient was fitted with a new prosthetic socket followed by multiple prosthetic adjustments without any change in symptoms. Shortly after symptom onset, the patient was initially trialed on escalating doses of gabapentin and, later, tizanidine; however, both medications failed to provide symptomatic relief and hence were discontinued. The patient's medication was then switched to pramipexole, which was also unsuccessful in improving myoclonus frequency (Table 1). As a result, the use of the prosthesis continued to be significantly limited.

**Table 1:** Details of medications prescribed to reduce myoclonus frequency in hamstrings and quadriceps

Medication and dosage	Reduction in myoclonus frequency?	Duration of medication course
Gabapentin, escalated up to 600 mg TID	No	4 weeks
Tizanidine, escalated up to 4 mg daily	No	2 months
Pramipexole, escalated up to 0.25 mg daily	No	4 weeks
Diagnostic right sciatic nerve block (mixture of 4 mL 2% lidocaine and 5 mL bupivacaine was injected)	Yes	Once
Right biceps femoris motor point block	Yes	Once

The patient was then prescribed a daily home knee stretching program to improve ROM about two months after symptom onset as recommended by a physiatrist. She had already been prescribed gabapentin and tizanidine at this time. The stretching protocol included maximally tolerated knee extension exercises to be performed three or more times per day. A more detailed description of the home exercise program recommended is presented in Table 2. The patient initially had a mild reduction in myoclonus severity a few weeks after the initiation of the home stretching program. She continued this home program for at least four months.

**Table 2:** Home knee extension stretch program recommended for the patient All of these activities have been generally recommended in the literature on patients with transtibial amputations. In addition, the patient was advised to continue to work on the stretches as long as they were not limited by pain and/or myoclonus

Name of stretch	Description
Quadriceps set	Advised to lie supine and press posterior right knee down onto bed or floor until stretch or tightness of quadriceps was felt. Advised to hold for 15-20 seconds. Perform 3 sets 3 times per day as tolerated
Sitting knee extension	Advised to sit in a chair, lift and straighten residual limb and hold stretch for 5-10 seconds. Perform 3 sets 3 times per day as tolerated.
Short arc quad	Advised to lie supine and place a large towel roll or a foam roll under the leg, straighten the knee, and hold for 10-15 seconds. Perform 3 sets 3 times per day as tolerated

A surface and needle electromyography (EMG) was performed after four months of symptom onset. At rest, electrical activity was unremarkable for vastus medialis, vastus lateralis, semitendinosus, semimembranosus, and both heads of the biceps femoris. However, during active and passive knee extension, both the anterior and posterior thigh muscles were activated. In an attempt to reduce the frequency of the myoclonus, lidocaine (5 ml, 1%) was injected in equal amounts into four locations in the proximal leg-patella tendon, quadriceps tendon, biceps femoris tendon, and semitendinosus muscle/tendon junction. Following the injections, the onset of the myoclonus was delayed until the knee was extended to about 20 degrees of flexion, and extension ROM increased up to 5 degrees of flexion.

Given the success of injections, a diagnostic right sciatic nerve block using electrical stimulation guidance was performed. A mixture of lidocaine (4 mL, 2%) and bupivacaine (5 mL) was injected when the maximal contraction was elicited at 1 milliamp in two separate portions with at least a one-minute pause in between to ensure a complete block. Immediately following the right sciatic nerve injection, the right biceps femoris remained taut and active on surface EMG. It was decided to additionally perform a biceps femoris motor point block. Following the motor point block, active knee extension to 10 degrees of flexion did not result in any spasms or myoclonus. Full extension, however, elicited co-contraction of knee flexors and extensors, though the magnitude of limb movement and surface EMG signal significantly decreased.

About two months after the diagnostic nerve block, the patient’s myoclonus symptoms had significantly decreased in frequency with marked improvement in knee extension ROM. She continued with her daily home knee stretching program during this period. Subsequently, she was refitted with a new prosthetic limb and was eventually able to ambulate with a nearly symmetrical gait for the first time since symptom onset. The patient was referred for chemodenervation using botulinum toxin A for long-term relief; however, she was unable to obtain insurance authorization for the procedure.

## Discussion

To our knowledge, this is the first reported case of "Jumping Stump" syndrome following a TTA revision surgery. Although prior case reports have documented varying timeframes regarding symptom onset of "Jumping Stump" syndrome, ranging from one month to up to three years postoperatively, the revision surgery appears to be the inciting event. Neuromas in the residual limb have been implicated in prior case reports as a potential ectopic trigger zone, leading to myoclonus or dystonia [3,4,5]. Our patient had a deep peroneal neuroma shortly after the initial TTA; however, it was excised, resulting in partial relief of stump pain months prior to symptom onset. The revision amputation surgery, which included myodesis and targeted muscle reinnervation of the right deep peroneal nerve, may have been the inciting etiology given that the symptom onset occurred only a few weeks postoperatively.

In our patient, needle EMG activity was unremarkable for muscles of the thigh at rest; however, during either active or passive knee extension, the posterior thigh muscles demonstrated a pattern of activation at approximately 40 degrees of knee flexion. The electrical activity became progressively more violent and, eventually, myoclonus-like activity of the quadriceps muscles would occur. It is suspected that the rapid firing of the hamstring muscles activated the patella stretch receptor, leading to afferent signaling to the spinal cord followed by efferent stimulation (and activation) of the quadriceps muscles. We suspect that the posterior thigh muscles were the primary initiator of the myoclonus in our patient.

Oral pharmacologic treatments were ineffective for symptom management for our patient. Escalating doses of gabapentin, tizanidine, and pramipexole did not reduce myoclonus frequency or duration. Prior case reports have documented some symptom relief from pramipexole, although this was not the case in our patient [7]. Further research is needed to better understand the pathophysiology of "Jumping Stump" syndrome, as well as to determine effective oral agents for symptomatic relief.

Our patient, however, did have some improvement in her symptoms following lidocaine injections to multiple muscles of the thigh, and then significant relief following the combination of a diagnostic sciatic nerve block and bicep femoris motor point block. The latter resulted in an immediate reduction in myoclonus frequency and improvement in ROM, up to 10 degrees of knee flexion. Throughout the various interventions, the patient was continued on a home knee extension stretch and ROM program. Ultimately, this resulted in a significant reduction in myoclonus frequency, allowing her to be fitted with a new prosthetic limb and return to prosthetic ambulation, essentially symptom-free.

Although the diagnostic nerve and motor block provided symptomatic relief, given the short-half life of lidocaine and bupivacaine, this was likely short-term. It is likely that her long-term symptomatic improvement was multifactorial in terms of its etiology, assumed to be primarily related to desensitization of a muscle-tendon stretch response. Thus, it is likely that the continued home knee extension stretch program also helped facilitate this sustained improvement. In addition, there may be a time component to symptom improvement also given it had been more than six months since the initial onset. However, because the diagnostic nerve and motor block allowed for temporary increased knee ROM with delayed myoclonus onset, it is postulated that chemodenervation with botulinum toxin combined with a home stretch program would have accelerated the improvement of symptoms, enabling a faster return of functional prosthetic limb use.

Although the patient was unable to obtain insurance authorization for botulinum toxin A injection, chemodenervation with botulinum toxin, as seen in previous case reports [6], remains a promising treatment modality for "Jumping Stump" syndrome. Given our patient's response to the diagnostic nerve and motor block, as well as improvement over many months with a consistent home stretching program, future studies should further investigate the role of chemodenervation in the management of "Jumping Stump" syndrome.

## Conclusions

"Jumping Stump" syndrome is a rare complication seen postoperatively in amputees and associated with significant psychosocial distress and functional impairment. Oral pharmacologic interventions were ineffective in our patient; however, a diagnostic nerve and motor block provided significant but temporary symptomatic relief. A consistent home knee extension stretching program over four months resulted in a sustained reduction in myoclonus frequency and improvement in knee ROM, allowing our patient to return to ambulation with a newly fitted prosthetic limb. Given the significant improvement in symptoms, chemodenervation combined with a home stretching program may be a promising treatment modality for "Jumping Stump" syndrome and should be further investigated.

## References

[1] Tyvaert L, Krystkowiak P, Cassim F, Houdayer E, Kreisler A, Destée A, Defebvre L (2009). Myoclonus of peripheral origin: two case reports. Mov Disord.

[2] Rombauts M, Duinslaeger E, Peers K, Kiekens C (2022). Jumping stump phenomenon: a case report. Prosthet Orthot Int.

[3] Hernández López M, Puentes Gutiérrez AB, López Zarzuela MC, García Bascones M (2023). Involuntary movements of the stump after transtibial amputation: Jumping stump syndrome (Article in Spanish). Rehabilitacion (Madr).

[4] Buntragulpoontawee M, Pattamapaspong N, Tongprasert S (2016). Multiple neuromas cause painful "Jumping Stump" in a transfemoral amputee: a case report. Int J Low Extrem Wounds.

[5] Giray E, Atalay KG, Şirazi S, Alp M, Yagci I (2021). An ultrasonographic and electromyographic evaluation of jumping stump possibly due to a neuroma in a patient with transradial amputation: a case report. J Back Musculoskelet Rehabil.

[6] Briand MM, Boudier-Réveret M, Rodrigue X, Sirois G, Chang MC (2020). A moving residual limb: botulinum toxin to the rescue. Transl Neurosci.

[7] Seidel S, Kechvar-Parast J, Sycha T, Zeitlhofer J (2011). The first case of a 'jumping stump' syndrome in a lower limb amputee responding to pramipexole. Eur J Neurol.

